# Are There Common Characteristics and Adjustment Processes among Gen Z?

**DOI:** 10.21315/mjms-12-2024-974

**Published:** 2025-02-28

**Authors:** Akiko Takeuchi, Shigeki Jin, Takayuki Kiuchi, Manabu Murakami

**Affiliations:** 1 Department of Forensic Medicine, Graduate School of Medicine, Hokkaido University, Sapporo, Hokkaido, Japan; 2 Department of Medical Education and General Medicine, Graduate School of Medicine, Hokkaido University, Sapporo, Hokkaido, Japan

Dear Editor,

The authors are interested in how Gen Z medical students adapt to their learning environments and were intrigued by a 2022 article by Zhuhra et al., which examined the process and contributing factors of college admissions among first-year Indonesian students (1). Although the study methodologies and settings are completely different, the authors’ research findings overlap with the results identified in the study by Zhuhra et al. (1), enhancing the generalizability of their conclusions.

The authors interviewed 22 first-year (liberal arts) and 11 third-year (preclinical training) students about their perceptions of the characteristics of their generation that affect their adaptation process, particularly the differences between them and previous generations. Each interview took approximately 60 min, and the interviews were recorded, converted into transcripts, analysed through coding, and inductively categorised based on similar content. The authors identified five categories: i) accustomed to digital devices and reliant on social media for information; ii) preferring flexibility and avoiding organisational discipline; iii) resistance to face-to-face communication despite disliking fear of missing out (FOMO); iv) preferring outcome-oriented and real-time feedback with good time management; and v) pragmatic thinking, emphasising work-life balance over remuneration or career advancement (Table 1).

Zhuhra et al. described Gen Z as “goaloriented, self-reliant, pragmatic,” and “at risk for FOMO” (1), which aligns with the authors’ abovementioned findings. Another study on burnout among medical students in Malaysia (2) suggested that the educational impact of high internet connectivity and protective parental environments on Gen Z may be universal. Since information can be quickly searched for on social media, Gen Z tends to value outcomes and dislike ambiguous processes (3, 4); however, they also have poor information literacy regarding credibility (3, 5).

The authors agree with the conceptual diagram by Zhuhra et al. of the adjustment process (1). While parents and seniors strongly encourage their children to become doctors (1, 2), a characteristic of Gen Z members may be their difficulty deciding whether they want to join the medical community of their own volition and follow its rules (3).

## Figures and Tables

**Table 1:** Summary of students’ perceptions of their generational characteristics

No.	Extracted categories	Categories’ explanation	Comments’ examples stated by the students
1.	Accustomed to digital devices and reliant on social media for information	Students are naturally accustomed to digital devices and use social media to look up information, yet the overwhelming amount of information and poor literacy limit their understanding.	“For our generation, staying connected with friends through LINE, Twitter (X), and Instagram is totally normal. We’re becoming more and more reliant on social media. The younger generations have even less media literacy and are quick to post videos without much thought.”
2.	Preferring flexibility and avoiding organisational discipline	Students are looking for a flexible working style and loose membership in an organisation, avoiding close ties with seniors, and are interested in becoming entrepreneurs independently.	“Since we can find a decent amount of information on our own, it feels like we’re doing more self-research instead of being taught directly by seniors. This is affecting teacher-student and senpai-kohai (senior-junior) relationships, making those connections weaker, and there’s a growing trend of people doing things more independently.”
3.	Resistance to face-to-face communication despite disliking fear of missing out (FOMO)	Students are introverted and prefer to be individualistic, avoiding face-to-face communication with others, while still wanting to remain constantly connected to their peers via the internet due to FOMO.	“I’m the kind of person who doesn’t study well alone, so I set up an environment where I can stay connected with my friends by studying over video calls. I don’t actually talk, and I keep my mic muted, but they can still see me studying through the webcam. Knowing that someone’s watching makes me want to stay focused and not slack off.”
4.	Preferring outcome-oriented and real-time feedback with good time performance	Students value time performance, prefer simple outcomes over complex processes, want timely evaluation feedback, and require a tailored teaching style that incorporates active learning techniques.	“If you don’t put effort into the exam questions or stick to the guidelines when answering, you won’t be sure if there’s a clear right answer, and stressing over it will waste a lot of time. I think it’s enough to just understand that you need to know the basics. Also, please make the answers public.”
5.	Pragmatic thinking, emphasising work-life balance over money or career advancement	Students choose careers suited to their abilities with realistic, passive thinking, prioritise personal life over work, and see little value in earning more than a minimum salary or pursuing career advancement.	“I chose my career in medicine with the somewhat shallow idea that it would be stable. I just want to earn enough money to live comfortably in the future. Rather than becoming a surgeon and taking on the risks of performing difficult operations, I’d prefer to work in ophthalmology, dermatology, or cosmetic surgery, where the risks are lower and the returns are higher.”
